# *catena*-Poly[[[diaqua­cobalt(II)]-μ-(3,5-dinitro-2-oxidobenzoato)-κ^3^ *O* ^1^,*O* ^2^:*O* ^1′^-[tetra­aqua­cobalt(II)]-μ-(3,5-dinitro-2-oxidobenzoato)-κ^3^ *O* ^1^:*O* ^1′^,*O* ^2^] dihydrate]

**DOI:** 10.1107/S1600536810052694

**Published:** 2010-12-24

**Authors:** Graham Smith, Urs D. Wermuth

**Affiliations:** a Faculty of Science and Technology, Queensland University of Technology, GPO Box 2434, Brisbane, Queensland 4001, Australia; b School of Biomolecular and Physical Sciences, Griffith University, Nathan, Queensland 4111, Australia

## Abstract

In polymeric title compound, {[Co_2_(C_7_H_2_N_2_O_7_)_2_(H_2_O)_6_]·2H_2_O}_*n*_, obtained from the reaction of 3,5-dinitro­salicylic acid with cobalt(II) acetate, both Co^II^ atoms are located on inversion centres and exhibit a distorted octahedral coordination geometry. The coordin­ation sphere about one Co^II^ atom comprises four O-atom donors from two bidentate chelate (O_phenolate_ and O_carbox­yl_) and bridging dianionic ligands and two water mol­ecules [Co—O range = 2.0249 (11)–2.1386 (14) Å], while that about the second Co^II^ atom has four water mol­ecules and two bridging carboxyl­ate O-donor atoms [Co—O range = 2.0690 (14)–2.1364 (11) Å]. The coordinated water mol­ecules as well as the water mol­ecules of solvation give O—H⋯O water–water and water–carboxyl hydrogen-bonding inter­actions in the three-dimensional framework structure.

## Related literature

For the structures of similar hydrated complexes of Co^II^, see: Deng *et al.* (2008 ▶); Sobolev *et al.* (2003 ▶); Tahir *et al.* (1996 ▶, 1997 ▶). For the structure of a mixed-ligand Co^II^ complex with 3,5-dinitro­salicylic acid and the structures of the acid and its salts, see: Zhong *et al.* (2009 ▶); Kumar *et al.* (1999 ▶); Smith *et al.* (2003 ▶, 2007 ▶). 
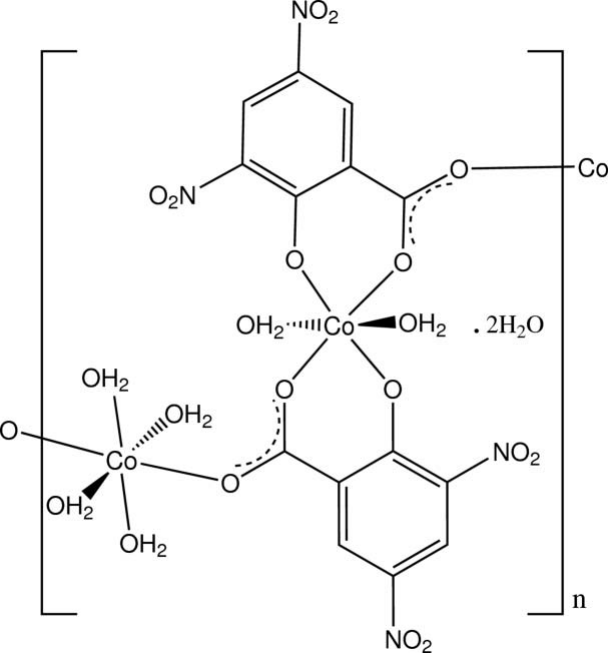


## Experimental

### 

#### Crystal data

[Co_2_(C_7_H_2_N_2_O_7_)_2_(H_2_O)_6_]·2H_2_O
*M* *_r_* = 714.20
Triclinic, 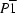

*a* = 6.8188 (3) Å
*b* = 7.7366 (4) Å
*c* = 11.3671 (5) Å
α = 92.658 (4)°
β = 96.313 (4)°
γ = 94.515 (4)°
*V* = 593.26 (5) Å^3^
*Z* = 1
Mo *K*α radiation
μ = 1.52 mm^−1^
*T* = 200 K
0.30 × 0.30 × 0.18 mm

#### Data collection

Oxford Diffraction Gemini-S Ultra CCD-detector diffractometer
Absorption correction: multi-scan (*CrysAlis PRO*; Oxford Diffraction, 2010 ▶) *T*_min_ = 0.865, *T*_max_ = 0.980
7532 measured reflections
2560 independent reflections
2236 reflections with *I* > 2σ(*I*)
*R*_int_ = 0.020

#### Refinement

*R*[*F* ^2^ > 2σ(*F* ^2^)] = 0.023
*wR*(*F* ^2^) = 0.061
*S* = 1.07
2560 reflections
225 parameters
H atoms treated by a mixture of independent and constrained refinement
Δρ_max_ = 0.32 e Å^−3^
Δρ_min_ = −0.47 e Å^−3^

### 

Data collection: *CrysAlis PRO* (Oxford Diffraction, 2010 ▶); cell refinement: *CrysAlis PRO*; data reduction: *CrysAlis PRO*; program(s) used to solve structure: *SIR92* (Altomare *et al.*, 1994 ▶); program(s) used to refine structure: *SHELXL97* (Sheldrick, 2008 ▶); molecular graphics: *PLATON* (Spek, 2009 ▶); software used to prepare material for publication: *PLATON*.

## Supplementary Material

Crystal structure: contains datablocks global, I. DOI: 10.1107/S1600536810052694/rn2077sup1.cif

Structure factors: contains datablocks I. DOI: 10.1107/S1600536810052694/rn2077Isup2.hkl

Additional supplementary materials:  crystallographic information; 3D view; checkCIF report

## Figures and Tables

**Table 1:** Hydrogen-bond geometry (Å, °)

*D*—H⋯*A*	*D*—H	H⋯*A*	*D*⋯*A*	*D*—H⋯*A*
O1*W*—H11*W*⋯O2*W*^i^	0.79 (3)	2.13 (3)	2.918 (2)	175 (2)
O1*W*—H12*W*⋯O4*W*	0.76 (3)	2.11 (3)	2.844 (2)	163 (3)
O2*W*—H21*W*⋯O2^ii^	0.75 (3)	2.08 (3)	2.7837 (18)	158 (3)
O2*W*—H22*W*⋯O51^iii^	0.78 (3)	2.21 (3)	2.8962 (19)	146 (3)
O3*W*—H31*W*⋯O12^iv^	0.84 (3)	1.94 (3)	2.6666 (19)	145 (2)
O3*W*—H32*W*⋯O4*W*^v^	0.72 (3)	2.31 (3)	2.927 (2)	145 (3)
O4*W*—H41*W*⋯O11^vi^	0.77 (3)	2.18 (3)	2.851 (2)	146 (2)
O4*W*—H42*W*⋯O32^vii^	0.74 (3)	2.51 (3)	3.178 (2)	152 (3)

Symmetry codes: (i) 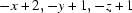; (ii) 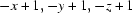; (iii) 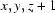; (iv) 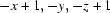; (v) 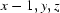; (vi) 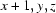; (vii) 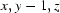.

**Fig. 1.**
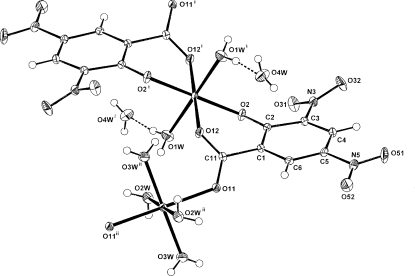
The molecular configuration and atom-numbering scheme for (I), with non-H atoms drawn as 40% probability ellipsoids. Both Co1 and Co2 lie on crystallographic inversion centres. For symmetry codes: (i) and (ii), see Table 1.

**Fig. 2.**
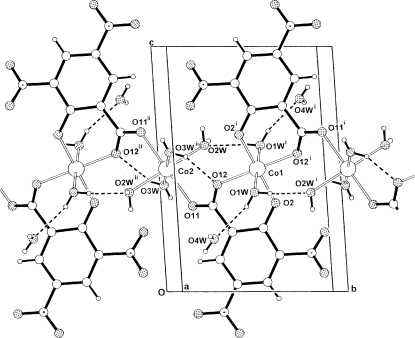
The coordination polymer structure of (I) extending across the b cell direction showing intra-unit hydrogen-bonding associations as dashed lines.

## supplementary crystallographic information

### Comment

3,5-Dinitrosalicylic acid (DNSA) has proved to be a useful synthon in crystal engineering (Kumar *et al.*, 1999) and the structures of a large number of its proton-transfer compounds with Lewis bases have been reported (Smith *et al.*, 2003, 2007). However, the structures of the transition metal complexes of DNSA are not so common and in particular, with Co^II^, there is only one example, a monomeric mixed-ligand complex with 2,2'-bipyridine (Zhong *et al.* , 2009), in which the DNSA ligand is dianionic and chelates through carboxyl and phenolate O donors. We obtained the title compound, having an empirical formula [Co(DNSA)(H_2_O)_4_], from the reaction of cobalt(II) acetate with 3,5-dinitrosalicylic acid in aqueous ethanol. This Co^II^ complex might have been expected to be typically octahedral and have a simple monomeric molecular formula involving the dianionic DNSA ligand in a bidentate chelate form, such as found in other similar hydrated cobalt(II) carboxylates, *e.g.* the acetate (Sobolev *et al.*, 2003), the 4-nitrosalicylate (Tahir *et al.*, 1997), the 4-formylbenzoate (Deng *et al.*, 2008) or the 3,5-dinitrobenzoate (Tahir *et al.*, 1996). However, the structure of (I) reported here showed the presence of a polymeric complex hydrate, {[Co_2_(C_7_H_2_N_2_O_7_)_2_(H_2_O)_6_]. 2H_2_O}_n_ (I), based on two slightly distorted octahedral but different Co^II^ centres.

In the structure (Fig. 1), the two separate six-coordinate CoO_6_ complex centres lie on crystallographic inversion centres at (1, 1/2, 1/2) (Co1) and (1/2, 0, 1/2) (Co2). The coordination sphere about Co1 comprises four O donors (O_phenolate_, O_carboxyl_) from two *trans*-related bidentate chelate dianionic DNSA ligands [Co—O, 2.0249 (11), 2.0508 (11) Å] and two water molecules [Co—O1*W*, 2.1386 (14) Å]. The second carboxyl O of each DNSA ligand (O11, O11^ii^) [for symmetry code (ii), see Table 1], provide *trans*-related bridges to the second Co centre [Co—O, 2.1364 (11) Å], with four water molecules (O2*W*, O3*W*) completing the coordination [Co—O, 2.1122 (14), 2.0690 (14) Å]. This results in polymer chain substructures which extend along the *b* cell direction (Fig. 2). The coordinated water molecules as well as the water molecule of solvation (O4*W*) give both water–water and inter-chain *O*—*H*···O_carboxyl, nitro_ hydrogen-bonding associations (Table 1), giving an overall three-dimensional framework structure.

### Experimental

The title compound was synthesized by heating together under reflux for 10 minutes, 1 mmol of cobalt(II) acetate and 2 mmol of 3,5-dinitrosalicylic acid in 50 ml of 50% ethanol–water. After concentration to *ca* 30 ml, partial room temperature evaporation of the hot-filtered solution gave large well formed red block crystals of (I).

### Refinement

Hydrogen atoms potentially involved in hydrogen-bonding interactions were located by difference methods and their positional and isotropic displacement parameters were refined. Other H atoms were included in the refinement in calculated positions with C–H = 0.93 Å and allowed to ride, with *U*_iso_(H) = 1.2*U*_eq_(C).

### Crystal data

**Table:**

[Co_2_(C_7_H_2_N_2_O_7_)_2_(H_2_O)_6_]·2H_2_O	*Z* = 1
*M**_r_* = 714.20	*F*(000) = 362
Triclinic, *P*1	*D*_x_ = 1.999 Mg m^−^^3^
Hall symbol: -P 1	Mo *K*α radiation, λ = 0.71073 Å
*a* = 6.8188 (3) Å	Cell parameters from 5528 reflections
*b* = 7.7366 (4) Å	θ = 3.3–28.7°
*c* = 11.3671 (5) Å	µ = 1.52 mm^−^^1^
α = 92.658 (4)°	*T* = 200 K
β = 96.313 (4)°	Plate, red
γ = 94.515 (4)°	0.30 × 0.30 × 0.18 mm
*V* = 593.26 (5) Å^3^	

### Data collection

**Table:**

Oxford Diffraction Gemini-S Ultra CCD-detector diffractometer	2560 independent reflections
Radiation source: fine-focus sealed tube	2236 reflections with *I* > 2σ(*I*)
graphite	*R*_int_ = 0.020
ω scans	θ_max_ = 27.0°, θ_min_ = 3.3°
Absorption correction: multi-scan (*CrysAlis PRO*; Oxford Diffraction, 2010)	*h* = −8→8
*T*_min_ = 0.865, *T*_max_ = 0.980	*k* = −9→9
7532 measured reflections	*l* = −14→14

### Refinement

**Table:**

Refinement on *F*^2^	Primary atom site location: structure-invariant direct methods
Least-squares matrix: full	Secondary atom site location: difference Fourier map
*R*[*F*^2^ > 2σ(*F*^2^)] = 0.023	Hydrogen site location: inferred from neighbouring sites
*wR*(*F*^2^) = 0.061	H atoms treated by a mixture of independent and constrained refinement
*S* = 1.07	*w* = 1/[σ^2^(*F*_o_^2^) + (0.0341*P*)^2^ + 0.1689*P*] where *P* = (*F*_o_^2^ + 2*F*_c_^2^)/3
2560 reflections	(Δ/σ)_max_ < 0.001
225 parameters	Δρ_max_ = 0.32 e Å^−^^3^
0 restraints	Δρ_min_ = −0.47 e Å^−^^3^

### Special details

**Table:**

Geometry. Bond distances, angles *etc*. have been calculated using the rounded fractional coordinates. All su's are estimated from the variances of the (full) variance-covariance matrix. The cell e.s.d.'s are taken into account in the estimation of distances, angles and torsion angles
Refinement. Refinement of *F*^2^ against ALL reflections. The weighted *R*-factor *wR* and goodness of fit *S* are based on *F*^2^, conventional *R*-factors *R* are based on *F*, with *F* set to zero for negative *F*^2^. The threshold expression of *F*^2^ > σ(*F*^2^) is used only for calculating *R*-factors(gt) *etc*. and is not relevant to the choice of reflections for refinement. *R*-factors based on *F*^2^ are statistically about twice as large as those based on *F*, and *R*- factors based on ALL data will be even larger.

### Fractional atomic coordinates and isotropic or equivalent isotropic displacement parameters (Å^2^)

**Table:**

	*x*	*y*	*z*	*U*_iso_*/*U*_eq_	
Co1	1.00000	0.50000	0.50000	0.0121 (1)	
Co2	0.50000	0.00000	0.50000	0.0130 (1)	
O1W	1.2442 (2)	0.45126 (19)	0.40373 (13)	0.0250 (4)	
O2	0.85747 (18)	0.60915 (14)	0.36011 (10)	0.0176 (3)	
O2W	0.4854 (2)	0.23727 (17)	0.59704 (13)	0.0210 (4)	
O3W	0.2062 (2)	0.0052 (2)	0.43256 (14)	0.0276 (4)	
O11	0.57060 (17)	0.13604 (14)	0.34878 (10)	0.0147 (3)	
O12	0.85493 (18)	0.26657 (14)	0.43617 (10)	0.0179 (3)	
O31	0.8352 (2)	0.89467 (15)	0.24132 (12)	0.0270 (4)	
O32	0.9601 (2)	0.86453 (16)	0.07589 (12)	0.0299 (4)	
O51	0.6887 (2)	0.35705 (18)	−0.17303 (11)	0.0335 (4)	
O52	0.5849 (2)	0.12456 (17)	−0.09262 (12)	0.0325 (4)	
N3	0.8741 (2)	0.80472 (17)	0.15690 (12)	0.0165 (4)	
N5	0.6572 (2)	0.2756 (2)	−0.08527 (13)	0.0208 (4)	
C1	0.7367 (2)	0.3493 (2)	0.24300 (14)	0.0125 (4)	
C2	0.8069 (2)	0.5305 (2)	0.25828 (14)	0.0121 (4)	
C3	0.8144 (2)	0.6185 (2)	0.15038 (14)	0.0133 (4)	
C4	0.7721 (2)	0.5375 (2)	0.03877 (14)	0.0154 (5)	
C5	0.7072 (2)	0.3626 (2)	0.03086 (14)	0.0153 (5)	
C6	0.6860 (2)	0.2692 (2)	0.13166 (14)	0.0139 (4)	
C11	0.7191 (2)	0.24360 (19)	0.35023 (14)	0.0122 (4)	
O4W	1.2050 (3)	0.1926 (2)	0.21463 (14)	0.0317 (5)	
H4	0.78670	0.59820	−0.02890	0.0180*	
H6	0.63760	0.15300	0.12370	0.0170*	
H11W	1.316 (4)	0.536 (4)	0.399 (2)	0.044 (8)*	
H12W	1.230 (4)	0.399 (4)	0.345 (3)	0.050 (8)*	
H21W	0.380 (5)	0.258 (4)	0.597 (3)	0.049 (9)*	
H22W	0.516 (5)	0.231 (4)	0.665 (3)	0.077 (11)*	
H31W	0.146 (4)	−0.086 (4)	0.451 (2)	0.050 (8)*	
H32W	0.158 (5)	0.044 (4)	0.382 (3)	0.058 (9)*	
H41W	1.310 (4)	0.161 (3)	0.224 (2)	0.045 (8)*	
H42W	1.148 (5)	0.140 (4)	0.165 (3)	0.070 (11)*	

### Atomic displacement parameters (Å^2^)

**Table:**

	*U*^11^	*U*^22^	*U*^33^	*U*^12^	*U*^13^	*U*^23^
Co1	0.0155 (2)	0.0109 (2)	0.0091 (2)	−0.0016 (1)	−0.0011 (1)	0.0015 (1)
Co2	0.0146 (2)	0.0127 (2)	0.0120 (2)	0.0006 (1)	0.0018 (1)	0.0030 (1)
O1W	0.0272 (7)	0.0233 (7)	0.0241 (7)	−0.0054 (6)	0.0098 (6)	−0.0056 (6)
O2	0.0282 (7)	0.0128 (5)	0.0106 (6)	0.0011 (5)	−0.0032 (5)	0.0007 (4)
O2W	0.0238 (7)	0.0198 (6)	0.0203 (7)	0.0034 (5)	0.0061 (6)	−0.0004 (5)
O3W	0.0180 (6)	0.0312 (8)	0.0339 (8)	−0.0007 (6)	−0.0009 (6)	0.0201 (7)
O11	0.0166 (6)	0.0136 (5)	0.0135 (6)	−0.0024 (4)	0.0011 (4)	0.0028 (4)
O12	0.0230 (6)	0.0147 (6)	0.0137 (6)	−0.0046 (5)	−0.0046 (5)	0.0042 (4)
O31	0.0455 (8)	0.0141 (6)	0.0228 (7)	0.0028 (6)	0.0109 (6)	−0.0007 (5)
O32	0.0448 (8)	0.0199 (6)	0.0272 (7)	−0.0054 (6)	0.0164 (6)	0.0084 (5)
O51	0.0532 (9)	0.0363 (8)	0.0093 (6)	−0.0044 (7)	0.0014 (6)	0.0025 (6)
O52	0.0432 (9)	0.0287 (7)	0.0214 (7)	−0.0152 (6)	0.0017 (6)	−0.0080 (6)
N3	0.0194 (7)	0.0139 (7)	0.0161 (7)	0.0002 (6)	0.0008 (6)	0.0049 (5)
N5	0.0220 (7)	0.0268 (8)	0.0123 (7)	−0.0005 (6)	−0.0002 (6)	−0.0025 (6)
C1	0.0122 (7)	0.0136 (7)	0.0118 (7)	0.0003 (6)	0.0019 (6)	0.0027 (6)
C2	0.0116 (7)	0.0132 (7)	0.0117 (7)	0.0016 (6)	0.0009 (6)	0.0023 (6)
C3	0.0149 (8)	0.0107 (7)	0.0144 (8)	0.0003 (6)	0.0017 (6)	0.0033 (6)
C4	0.0165 (8)	0.0185 (8)	0.0118 (8)	0.0021 (6)	0.0026 (6)	0.0047 (6)
C5	0.0160 (8)	0.0193 (8)	0.0098 (8)	0.0004 (6)	0.0000 (6)	−0.0019 (6)
C6	0.0135 (7)	0.0128 (7)	0.0149 (8)	−0.0008 (6)	0.0011 (6)	0.0010 (6)
C11	0.0163 (8)	0.0091 (7)	0.0116 (8)	0.0014 (6)	0.0030 (6)	0.0004 (6)
O4W	0.0266 (8)	0.0380 (9)	0.0288 (8)	0.0067 (7)	−0.0036 (6)	−0.0078 (7)

### Geometric parameters (Å, °)

**Table:**

Co1—O1W	2.1386 (14)	O1W—H12W	0.76 (3)
Co1—O2	2.0249 (11)	O1W—H11W	0.79 (3)
Co1—O12	2.0508 (11)	O2W—H21W	0.75 (3)
Co1—O1W^i^	2.1386 (14)	O2W—H22W	0.78 (3)
Co1—O2^i^	2.0249 (11)	O3W—H32W	0.72 (3)
Co1—O12^i^	2.0508 (11)	O3W—H31W	0.84 (3)
Co2—O2W	2.1122 (14)	O4W—H42W	0.74 (3)
Co2—O3W	2.0690 (14)	O4W—H41W	0.77 (3)
Co2—O11	2.1364 (11)	N3—C3	1.462 (2)
Co2—O2W^ii^	2.1122 (14)	N5—C5	1.447 (2)
Co2—O3W^ii^	2.0690 (14)	C1—C11	1.509 (2)
Co2—O11^ii^	2.1364 (11)	C1—C2	1.442 (2)
O2—C2	1.2817 (19)	C1—C6	1.379 (2)
O11—C11	1.2572 (18)	C2—C3	1.434 (2)
O12—C11	1.2660 (19)	C3—C4	1.379 (2)
O31—N3	1.2242 (19)	C4—C5	1.386 (2)
O32—N3	1.2335 (19)	C5—C6	1.397 (2)
O51—N5	1.234 (2)	C4—H4	0.9300
O52—N5	1.228 (2)	C6—H6	0.9300
			
O1W—Co1—O2	91.94 (5)	Co1—O1W—H12W	122 (2)
O1W—Co1—O12	90.72 (5)	H21W—O2W—H22W	101 (4)
O1W—Co1—O1W^i^	180.00	Co2—O2W—H21W	110 (2)
O1W—Co1—O2^i^	88.06 (5)	Co2—O2W—H22W	113 (2)
O1W—Co1—O12^i^	89.28 (5)	H31W—O3W—H32W	114 (3)
O2—Co1—O12	87.76 (4)	Co2—O3W—H31W	107.2 (19)
O1W^i^—Co1—O2	88.06 (5)	Co2—O3W—H32W	133 (3)
O2—Co1—O2^i^	180.00	H41W—O4W—H42W	108 (3)
O2—Co1—O12^i^	92.24 (4)	O31—N3—O32	122.86 (14)
O1W^i^—Co1—O12	89.28 (5)	O32—N3—C3	118.20 (13)
O2^i^—Co1—O12	92.24 (4)	O31—N3—C3	118.94 (13)
O12—Co1—O12^i^	180.00	O51—N5—C5	118.39 (14)
O1W^i^—Co1—O2^i^	91.94 (5)	O51—N5—O52	122.71 (15)
O1W^i^—Co1—O12^i^	90.72 (5)	O52—N5—C5	118.90 (14)
O2^i^—Co1—O12^i^	87.76 (4)	C2—C1—C11	119.84 (14)
O2W—Co2—O3W	89.80 (6)	C6—C1—C11	118.89 (14)
O2W—Co2—O11	90.74 (5)	C2—C1—C6	121.27 (14)
O2W—Co2—O2W^ii^	180.00	C1—C2—C3	114.96 (14)
O2W—Co2—O3W^ii^	90.20 (6)	O2—C2—C1	123.15 (14)
O2W—Co2—O11^ii^	89.26 (5)	O2—C2—C3	121.88 (14)
O3W—Co2—O11	86.59 (5)	C2—C3—C4	123.97 (14)
O2W^ii^—Co2—O3W	90.20 (6)	N3—C3—C2	119.02 (13)
O3W—Co2—O3W^ii^	180.00	N3—C3—C4	116.99 (14)
O3W—Co2—O11^ii^	93.41 (5)	C3—C4—C5	117.76 (14)
O2W^ii^—Co2—O11	89.26 (5)	N5—C5—C6	119.33 (14)
O3W^ii^—Co2—O11	93.41 (5)	C4—C5—C6	121.83 (15)
O11—Co2—O11^ii^	180.00	N5—C5—C4	118.84 (14)
O2W^ii^—Co2—O3W^ii^	89.80 (6)	C1—C6—C5	120.05 (14)
O2W^ii^—Co2—O11^ii^	90.74 (5)	O12—C11—C1	118.49 (13)
O3W^ii^—Co2—O11^ii^	86.59 (5)	O11—C11—O12	123.51 (14)
Co1—O2—C2	124.09 (10)	O11—C11—C1	118.00 (13)
Co2—O11—C11	123.33 (10)	C3—C4—H4	121.00
Co1—O12—C11	126.18 (10)	C5—C4—H4	121.00
H11W—O1W—H12W	110 (3)	C1—C6—H6	120.00
Co1—O1W—H11W	112.7 (19)	C5—C6—H6	120.00
			
O1W—Co1—O2—C2	59.16 (12)	O51—N5—C5—C6	−175.47 (14)
O12—Co1—O2—C2	−31.48 (12)	O52—N5—C5—C4	−174.28 (14)
O1W^i^—Co1—O2—C2	−120.84 (12)	O52—N5—C5—C6	4.8 (2)
O12^i^—Co1—O2—C2	148.52 (12)	C6—C1—C2—O2	−179.22 (14)
O1W—Co1—O12—C11	−99.19 (13)	C6—C1—C2—C3	1.4 (2)
O2—Co1—O12—C11	−7.27 (13)	C11—C1—C2—O2	0.3 (2)
O1W^i^—Co1—O12—C11	80.81 (13)	C11—C1—C2—C3	−179.01 (12)
O2^i^—Co1—O12—C11	172.73 (13)	C2—C1—C6—C5	1.7 (2)
O2W—Co2—O11—C11	58.56 (12)	C11—C1—C6—C5	−177.86 (13)
O3W—Co2—O11—C11	148.31 (12)	C2—C1—C11—O11	139.77 (14)
O2W^ii^—Co2—O11—C11	−121.44 (12)	C2—C1—C11—O12	−40.9 (2)
O3W^ii^—Co2—O11—C11	−31.69 (12)	C6—C1—C11—O11	−40.7 (2)
Co1—O2—C2—C1	37.23 (19)	C6—C1—C11—O12	138.71 (14)
Co1—O2—C2—C3	−143.47 (11)	O2—C2—C3—N3	−2.4 (2)
Co2—O11—C11—O12	9.1 (2)	O2—C2—C3—C4	176.25 (14)
Co2—O11—C11—C1	−171.53 (10)	C1—C2—C3—N3	176.94 (12)
Co1—O12—C11—O11	−141.37 (12)	C1—C2—C3—C4	−4.4 (2)
Co1—O12—C11—C1	39.30 (19)	N3—C3—C4—C5	−177.33 (13)
O31—N3—C3—C2	−31.2 (2)	C2—C3—C4—C5	4.0 (2)
O31—N3—C3—C4	150.07 (14)	C3—C4—C5—N5	178.51 (13)
O32—N3—C3—C2	149.57 (14)	C3—C4—C5—C6	−0.5 (2)
O32—N3—C3—C4	−29.2 (2)	N5—C5—C6—C1	178.72 (13)
O51—N5—C5—C4	5.5 (2)	C4—C5—C6—C1	−2.3 (2)

Symmetry codes: (i) −*x*+2, −*y*+1, −*z*+1; (ii) −*x*+1, −*y*, −*z*+1.

### Hydrogen-bond geometry (Å, °)

**Table:**

*D*—H···*A*	*D*—H	H···*A*	*D*···*A*	*D*—H···*A*
O1W—H11W···O2W^i^	0.79 (3)	2.13 (3)	2.918 (2)	175 (2)
O1W—H12W···O4W	0.76 (3)	2.11 (3)	2.844 (2)	163 (3)
O2W—H21W···O2^iii^	0.75 (3)	2.08 (3)	2.7837 (18)	158 (3)
O2W—H22W···O51^iv^	0.78 (3)	2.21 (3)	2.8962 (19)	146 (3)
O3W—H31W···O12^ii^	0.84 (3)	1.94 (3)	2.6666 (19)	145 (2)
O3W—H32W···O4W^v^	0.72 (3)	2.31 (3)	2.927 (2)	145 (3)
O4W—H41W···O11^vi^	0.77 (3)	2.18 (3)	2.851 (2)	146 (2)
O4W—H42W···O32^vii^	0.74 (3)	2.51 (3)	3.178 (2)	152 (3)
C6—H6···O52^viii^	0.93	2.52	3.420 (2)	164

Symmetry codes: (i) −*x*+2, −*y*+1, −*z*+1; (iii) −*x*+1, −*y*+1, −*z*+1; (iv) *x*, *y*, *z*+1; (ii) −*x*+1, −*y*, −*z*+1; (v) *x*−1, *y*, *z*; (vi) *x*+1, *y*, *z*; (vii) *x*, *y*−1, *z*; (viii) −*x*+1, −*y*, −*z*.
